# Supplementary figures and images for: Bio-guided isolation of a new sesquiterpene from Artemisia cina with anthelmintic activity against Haemonchus contortus L3 infective larvae
Source: PLoS One. 2024 Jun 12;19(6):e0305155. doi: 10.1371/journal.pone.0305155 (PMC11168668; doi:10.1371/journal.pone.0305155)

**
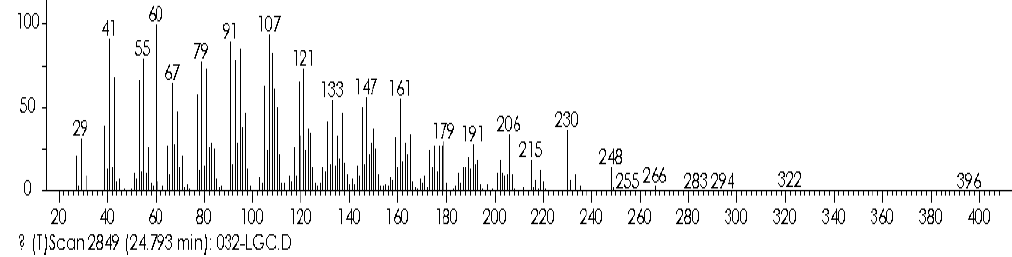
**

**S1 Fig. Mass spectra of cinic acid obtained by CG-MS.**

Supplement: S1 Fig — (DOCX) [file pone.0305155.s001.docx]
