# Supplementary material for: Bio-guided isolation of a new sesquiterpene from Artemisia cina with anthelmintic activity against Haemonchus contortus L3 infective larvae
Source: PLoS One. 2024 Jun 12;19(6):e0305155. doi: 10.1371/journal.pone.0305155 (PMC11168668; doi:10.1371/journal.pone.0305155)
Supplement: S5 Fig — (DOCX) [file pone.0305155.s005.docx]

**S5 Fig. HMBC spectra of cinic acid dissolved in CD_3_COCD_3_ and obtained at 500 MHz.**
