# Supplementary material for: Bio-guided isolation of a new sesquiterpene from Artemisia cina with anthelmintic activity against Haemonchus contortus L3 infective larvae
Source: PLoS One. 2024 Jun 12;19(6):e0305155. doi: 10.1371/journal.pone.0305155 (PMC11168668; doi:10.1371/journal.pone.0305155)
Supplement: S6 Fig — COSY experiment of cinic acid dissolved in CD3COCD3 and obtained at 500 MHz: a) COSY spectra of cinic acid and b) COSY spectra of the correlation between H7 (δ 2.85 m, br) and H8 (δ 4.27 m, br), consistent with the cis-orientation of the protons. (DOCX) [file pone.0305155.s006.docx]

|  |
| --- |
| **a)** |
|  |
| **b)** |
| **S6 Fig. COSY experiment of cinic acid dissolved in CD_3_COCD_3_ and obtained at 500 MHz a) COSY spectra of cinic acid and b) COSY spectra of the correlation between H7 (δ 2.85 m, br) and H8 (δ 4.27 m, br) consistent with *cis*-orientation of the protons.** |
