# Supplementary material for: Bio-guided isolation of a new sesquiterpene from Artemisia cina with anthelmintic activity against Haemonchus contortus L3 infective larvae
Source: PLoS One. 2024 Jun 12;19(6):e0305155. doi: 10.1371/journal.pone.0305155 (PMC11168668; doi:10.1371/journal.pone.0305155)
Supplement: S7 Fig — NOESY experiment of cinic acid dissolved in CD3COCD3 and obtained at 500 MHz: a) NOESY spectra of cinic acid and b) NOESY spectra of the correlation between the two methyl groups (δ 1.20 d, J = 6.83 Hz) and (δ 1.06 s)), consistent with the cis-orientation of the protons. (DOCX) [file pone.0305155.s007.docx]

|  |
| --- |
| **a)** |
|  |
| **b)** |
| **S7 Fig. NOESY experiment of cinic acid dissolved in CD_3_COCD_3_ and obtained at 500 MHz a) NOESY spectra of cinic acid and b) NOESY spectra of the correlation between the two methyl groups (δ 1.20 d, J= 6.83 Hz) and (δ 1.06 s)) consistent with *cis*-orientation of the protons.** |
